# Astrocyte IP3R2-dependent Ca^2+^ signaling is not a major modulator of neuronal pathways governing behavior

**DOI:** 10.3389/fnbeh.2014.00384

**Published:** 2014-11-12

**Authors:** Jeremy Petravicz, Kristen M. Boyt, Ken D. McCarthy

**Affiliations:** ^1^Curriculum in Neurobiology, University of North Carolina at Chapel HillChapel Hill, NC, USA; ^2^Department of Pharmacology, University of North Carolina at Chapel HillChapel Hill, NC, USA

**Keywords:** astrocyte, calcium, behavior, IP3 receptor, gliotransmission

## Abstract

Calcium-dependent release of gliotransmitters by astrocytes is reported to play a critical role in synaptic transmission and be necessary for long-term potentiation (LTP), long-term depression (LTD) and other forms of synaptic modulation that are correlates of learning and memory. Further, physiological processes reported to be dependent on Ca^2+^ fluxes in astrocytes include functional hyperemia, sleep, and regulation of breathing. The preponderance of findings indicate that most, if not all, receptor dependent Ca^2+^ fluxes within astrocytes are due to release of Ca^2+^ through IP3 receptor/channels in the endoplasmic reticulum. Findings from several laboratories indicate that astrocytes only express IP3 receptor type 2 (IP3R2) and that a knockout of IP3R2 obliterates the GPCR-dependent astrocytic Ca^2+^ responses. Assuming that astrocytic Ca^2+^ fluxes play a critical role in synaptic physiology, it would be predicted that elimination of astrocytic Ca^2+^ fluxes would lead to marked changes in behavioral tests. Here, we tested this hypothesis by conducting a broad series of behavioral tests that recruited multiple brain regions, on an IP3R2 conditional knockout mouse model. We present the novel finding that behavioral processes are unaffected by lack of astrocyte IP3R-mediated Ca^2+^ signals. IP3R2 cKO animals display no change in anxiety or depressive behaviors, and no alteration to motor and sensory function. Morris water maze testing, a behavioral correlate of learning and memory, was unaffected by lack of astrocyte IP3R2-mediated Ca^2+^-signaling. Therefore, in contrast to the prevailing literature, we find that neither receptor-driven astrocyte Ca^2+^ fluxes nor, by extension, gliotransmission is likely to be a major modulating force on the physiological processes underlying behavior.

## Introduction

Astrocytes are a major population of brain cells and closely associate with all CNS cell types, particularly neurons. It is estimated astrocytes contact 50–90% of synapses in a given brain region (Oliet et al., 2001; Genoud et al., 2006) and, within the hippocampus ensheathe ~100,000 synapses (Bushong et al., 2002). Over the past decade, astrocytes have increasingly been viewed not only as supporting cells in the brain, but also as active partners in many of the mechanisms underlying brain physiology. A large literature indicates that astrocytes regulate synaptic transmission and plasticity through GPCR/IP3R-mediated, Ca^2+^-dependent release of molecules termed gliotransmitters (Agulhon et al., 2012). Gliotransmission has been reported to regulate both glutamatergic and GABAergic transmission (Fellin et al., 2004; Serrano et al., 2006; Perea and Araque, 2007; Benedetti et al., 2011; Min and Nevian, 2012). A critical and necessary component of this signaling pathway is the IP3R, of which astrocytes express solely IP3R2 (Sharp et al., 1999; Holtzclaw et al., 2002; Hertle and Yeckel, 2007; Petravicz et al., 2008). Astrocyte GPCR-mediated Ca^2+^ signaling (somatic and process-localized responses) in response to neuronal activity in acute slice preparations (Petravicz et al., 2008; Di Castro et al., 2011) and *in vivo* (Takata et al., 2011) are dependent on the activation of IP3R2. Alterations to synaptic plasticity and neuronal circuit function have been reported using a germ-line IP3R2 knockout (IP3R2 KO) mouse model (Navarrete et al., 2012; Wang et al., 2012; Perez-Alvarez et al., 2014). However, the results from studies using this model system are controversial, with an equal number of publications from our laboratory and other independent laboratories reporting no alterations in synaptic plasticity or other physiological processes hypothesized to be modulated by IP3R2-mediated Ca^2+^ dependent signaling (Fiacco et al., 2007; Petravicz et al., 2008; Agulhon et al., 2010; Wang et al., 2012; Nizar et al., 2013; Takata et al., 2013; Bonder and McCarthy, 2014). Therefore, the role of astrocytes as modulators of neuronal circuit function remains unresolved.

A behavioral approach to the question of whether astrocytes are integral components of neuronal circuit activity and the plasticity mechanisms underlying behavior is a major shift from the standard analysis of astrocyte neuron interactions; that is, electrophysiological studies using brain slices and *in vivo* Ca^2+^ imaging. We postulate that if astrocytic IP3R-mediated, Ca^2+^-dependent release of gliotransmitters is providing a significant source of modulation to neuronal circuit function and plasticity, it follows that lack of Ca^2+^ signaling in astrocytes would affect behaviors dependent on neuronal circuit activity and synaptic plasticity. This hypothesis has been shown valid for several other astrocytic pathways known to modulate synaptic transmission such as glutamate reuptake, gap junction communication and nitric oxide production (Frisch et al., 2003; Theis et al., 2003; Abu-Ghanem et al., 2008; Kiryk et al., 2008). In this report, we used a novel conditional knockout mouse model of the IP3R2 to selectively block IP3R-mediated Ca^2+^ signaling in response to neuronal activity from the majority (>80%) of CNS astrocytes. We performed a battery of behavioral tests to determine if lack of IP3R-mediated Ca^2+^ signaling in astrocytes affects behavior. We present the unexpected finding that lack of Ca^2+^ signaling in astrocytes results in no detectable alteration in any of the behaviors tested. Overall, these findings bring into question the physiological significance of gliotransmission in modulating neuronal circuits.

## Materials and methods

### Generation of IP3R2 cKO mice

IP3R2flox/flox mice on a C57BL/6 background were crossed to C57BL/6 mice expressing Cre-recombinase under a fragment of the human GFAP promoter (Casper and McCarthy, 2006) to generate mice heterozygous for the floxed IP3R2 allele and Cre-recombinase. These mice were then interbred with C57BL/6 mice homozygous for the floxed IP3R2 allele to generate mice heterozygous for Cre-recombinase and homozygous for the floxed IP3R2 allele. Mice from these breeding that were homozygous for the IP3R2 floxed allele and heterozygous for Cre-recombinase were designated as IP3R2 cKO. Mice homozygous for the IP3R2 floxed allele, but not carrying Cre-recombinase, were designated as control mice. Mice were genotyped by PCR analysis using genomic DNA and primers specific to Cre-recombinase and the floxed IP3R2 allele.

### Calcium imaging

Hippocampal slices were prepared as previously described (Petravicz et al., 2008) with the following modifications. Brains were sectioned in a modified slicing buffer containing the following in mM: 130 NaCl, 10 glucose, 1.25 NaH_2_PO_4_, 24 NaHCO_3_, 3.5 KCl, 5 MgCl_2_, and 1 CaCl_2_ and bubbled with 95% O2 and 5% CO_2_. Hippocampal slices were incubated at 35-37°C for 20 min in slicing buffer containing 1 μM SR101 which preferentially loads astrocytes (Nimmerjahn et al., 2004). Hippocampal slices were then transferred for 10 min to warm (35-37°C) ACSF containing the following in mM: 130 NaCl, 10 glucose, 1.25 NaH_2_PO_4_, 24 NaHCO_3_, 3.5 KCl, 2.5 MgCl_2_, and 1.5 CaCl_2_ and bubbled with 95% O_2_ and 5% CO_2_. The calcium indicator Oregon Green BAPTA-AM (OGB-1AM) was suspended in 100 μl ACSF containing 20% pluronic acid (final DMSO concentration 0.04%). The ACSF contained the following in mM: 150 NaCl, 2.5 KCl and 10 HEPES with the pH adjusted to 7.3-7.5 with 1M NaOH. Hippocampal slices were placed in a perfusion chamber with a constant flow of oxygenated normal ACSF. Pipettes (1–2 MΩ resistance) filled with the OGB-1AM containing ACSF were lowered to the surface of the slices and backpressure applied. The pipette was then lowered 40 μm into the hippocampal striatum proximal to the pyramidal cell layer of the CA1 or CA3 region. OGB-1AM was injected for 2–3 min (based on the pipette resistance) and then lowered a further 35 μm deeper into the slice and injected for an additional 2–3 min. The pipette was then removed and the slice transferred to room temperature (25°C) oxygenated ACSF and allowed to recover for a minimum of 45 min prior to imaging. Astrocyte calcium increases were recorded using a two photon imaging system (Coherent Chameleon Ultra, Coherent Inc, Santa Clara, CA). Astrocytes were identified by SR101 loading and regions of interest were drawn around the SR101 positive cell bodies. Increases in average fluorescence in regions of interest indicate increase in Ca^2+^ concentration. Fold increase over baseline was calculated for each trace and reported as ΔF/F_0_.

### Immunohistochemistry

For anti-IP3R2 and anti-GFAP immunohistochemistry, mice were perfused with PBS followed by 4% PFA, post-fixed in 4% PFA overnight, and 40-μm fixed slices were prepared using a vibrating microtome. The fixed slices were then blocked in 10% (vol/vol) normal goat serum with 1% (vol/vol) Triton in PBS (1 h at room temperature) and stained for GFAP and IP3R2 overnight (<4°C). The primary antibodies used were rabbit anti-IP3R2, (1:20, AB3000; Millipore) and mouse anti-GFAP (1:400, G3893; Sigma), whereas the secondary antibodies were Alexa Fluor 488 goat anti-rabbit (1:250, A11034; Invitrogen) and Alexa Fluor 633 goat anti-mouse (1:250, A21236; Invitrogen).

### Behavioral testing cohorts

Behavioral testing cohorts consisted of age matched IP3R2 cKO mice with littermate controls. Mice were tested between the ages of P60–80. The weights of the mice used in behavioral testing ranged from 21.2 to 26.9 g, and were not significantly different between control and IP3R2 cKO (data not shown). Mice were housed in a mixed population of control and mutant animals and segregated by sex. Mice were housed five to a cage and were placed in the experiment room for 20 min prior to testing for acclimatization of the mice to the testing environment.

### Elevated plus-maze test for anxiety-like behaviors

Mice were given one 5-min trial on the elevated plus-maze. The elevated plus-maze apparatus consisted of two open arms and two closed arms with 40 cm high walls. The maze is elevated 50 cm from the floor and the arms are 21 cm long. Mice were placed in the center area of the maze (9.5 × 9.5 cm), and allowed to freely explore the maze. Measures were taken of time in, and number of entries into, the open and closed arms. Percent open arm time was calculated as 100 × [open arm time/(open arm time + closed arm time)]. Percent open arm entries were calculated using the same formula, but using the measure for entries.

### Open field activity

Exploration in a novel environment was assessed by a 1 h trial in an open field (40 × 40 × 30 cm) crossed by a grid of photobeams (VersaMax system, AccuScan Instruments). Counts were taken of the number of photobeams broken during the trial in 5-min intervals, with separate measures for horizontal activity, fine movements (repeated breaking of the same set of photobeams), and vertical activity (rearing movements). Percent center time was calculated by dividing the time spent in the designated center region of the activity box divided by the total time for each 5-min interval.

### Rotarod

Mice were assessed for balance and motor coordination on an accelerating rotarod (Ugo-Basile, Stoelting Co., Wood Dale, IL). The revolutions per min were initially set at 3 rpm, and progressively increased to 30 rpm over the course of a 5 min trial session. Each mouse was given 5 trials in total, 3 trials on the first day and 2 trials 48 h later. Each trial was separated by 45 sec between trials. Latency to fall or rotate off the top of the turning barrel was measured by the rotarod timer. If the mouse immediately fell off at the beginning of the first trial, that trial was not counted, and the mouse was given a new trial.

### Acoustic startle response

The acoustic startle measure was based on the reflexive whole body flinch following exposure to a sudden noise. Animals were tested using the San Diego Instruments SR-Lab system using the procedure described by Crawley and Paylor (1997). Briefly, mice were placed in small Plexiglas cylinder within a large sound chamber (San Deigo Instruments). The cylinder is place upon a piezoelectric transducer, which allowed the vibration to be detected and quantified by computer software. The chamber includes a fan, a house light, and a loudspeaker for the acoustic stimuli consisting of bursts of white noise. Background sound levels were maintained at 70 dB. Each mouse was given one session consisting of 42 trials following a 5 min habituation period. Seven different types of trials were presented: no-stimulus trials, trials with the acoustic startle stimulus (40 ms, 120 dB) alone, and trials with a prepulse stimulus (20 ms, at 74, 78, 82, 86, or 90 dB) delivered 100 ms before the onset of the startle stimulus. The different trial types were presented in blocks of 7, in randomized order within each block, with an average interval of 15 s (range: 10–20 s). Measures were taken of the startle amplitude for each trial, defined as the peak response during a 65-ms sampling window that began with the onset of the startle stimulus. An overall analysis was performed for each subject's data for levels of prepulse inhibition at each prepulse sound level calculated as 100-[(responses amplitude for prepulse and startle stimulus together/response amplitude for the startle stimulus alone) × 100].

### Morris water maze

Mice were assessed for spatial learning using the Morris water maze. The water maze consisted of a large circular pool (diameter = 122 cm) partially filled with water (45 cm deep, 24-26°C) located in a room with numerous visual cues. Mice were tracked by an automated system (Noldus Ehtovision 3.0) using a camera suspended above the water maze pool. Mice were tested for their ability to find an escape platform (diameter = 12 cm) on three different components: visible platform acquisition, hidden (submerged) platform acquisition, and subsequent probe trial in the absence of the platform. Following the hidden platform probe trial, the platform was moved to a new location and the mice were trained again to find the platform and given a subsequent probe trial to measure reversal learning. In both hidden and reversal learning, the criteria for learning was an average group latency of 15 s or less to locate a platform across four consecutive trials per day. In the visible platform test, each animal was given four trials per day for 3 days to swim to an escape platform indicated by a patterned cylinder extended above the surface of the water on the hidden platform. For each trial the mouse was placed into the pool, at one of four possible locations determined randomly and given 60 s to find the visible platform. If the mouse found the platform, the trial ended and the mouse was allowed to remain on the platform for 10–15 s prior to the start of the next trial. If the mouse did not find the platform, the mouse was placed on the platform for 10–15 s and then given the next trial. Measures were taken of latency to find the platform, swimming distance, and swimming velocity by the Nodulus Ethovision tracking system. Mice were then trained on the hidden platform test. The same testing procedure as described above was used, with each animal receiving four trials per day. At the end of the day when the average group latency reached 15 s or less, mice were given a 1 min probe trial in the pool with the platform removed. In this probe trial, selective quadrant search was evaluated by measuring percent of time spent in each quadrant of the pool. Spatial learning was demonstrated by greater swim times in the quadrant where the platform had previously been present in comparison to other quadrants in the pool. Following 1–2 days after the hidden probe trial, mice were tested for reversal learning using the same training paradigm except that the platform was located in the diagonal opposite quadrant from its previous location. Measures were taken for latency to find the platform, swimming distance and swimming velocity. Upon reaching the 15 s or fewer criterions the platform was removed from the pool and the group was given the probe trial to evaluate reversal learning.

### Tail suspension test

Mice were assessed for depressive behaviors by measuring time spent immobile using the tail suspension test. Mice were suspended by the tail with tape for 5 min inside of a plastic, open faced box and their activity recorded. The total duration of immobility (defined as no struggling movements) for each mouse was manually scored. Percent time immobile was calculated by dividing the duration of immobility by the total duration of the trial. Percent immobile time was averaged among cohorts and reported as average ±s.e.m.

### Statistical analysis

All statistical analysis was completed using Graphpad Prism6. Data was analyzed in the following manner: For elevated plus maze and tail suspension test, unpaired Student *t*-tests. For rotarod, open field activity and acoustic startle response, repeated measures ANOVA. For Morris water maze visual, hidden and reversal of hidden training trials were tested with Two-Way repeated measures ANOVA. For hidden and reversal of hidden probe trials, each genotype was tested with One-Way repeated measures ANOVA to test for quadrant preference, and between genotypes with Two Way repeated measure ANOVA with Tukey's multiple comparisons *post-hoc* test for significance. Statistical differences of *p* < 0.05 were reported as significant. All data is reported as mean ± s.e.m.

## Results

### Lack of Ca^2+^ responses in IP3R2 cKO mice

**Figure 1:**
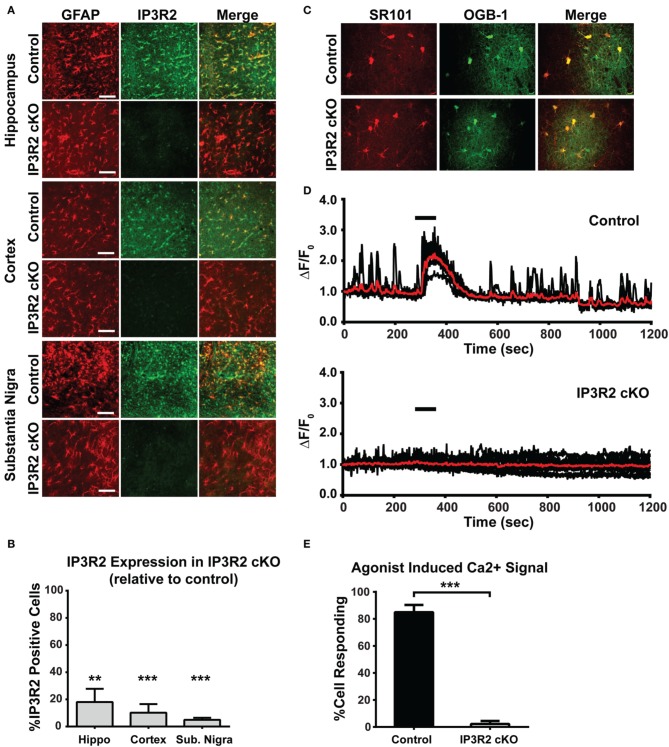
**Immunohistochemical and Ca^2+^ imaging analysis of IP3R2 recombination efficiency. (A)** Astrocytes labeled with GFAP (left column) display a high level of colocalization with IP3R2 staining (IP3R2 middle column; Merge with GFAP right column). Scale bar, 100 μm. **(B)** Quantification of three different brain regions for recombination efficiency in the IP3R2 cKO found high levels of recombination in the brain regions examined. Data are presented as GFAP positive cells colocalizing with IP3R2 relative to control (100% colocalization). **(C)** Two-photon images of hippocampal sections loaded with SR101 (left) and OGB-1AM (middle) and their colocalization (right). **(D)** Population Ca^2+^ traces from representative experiments (Control, *n* = 7 cells; IP3R2 cKO, *n* = 12 cells) showing responses to agonist application (black bar; coapplication of 10 μM DHPG, 10 μM carbachol, 10 μM histamine). Red trace represents cell population average for each experiment. **(E)** Quantification of agonist-induced Ca^2+^ increases in IP3R2 cKO hippocampal slices in all experiments (Control, *n* = 97 cells; IP3R2 cKO, *n* = 63). Error bars indicate s.e.m. ^**^ *p* < 0.01; ^***^ *p* < 0.001.

Our lab has previously reported that a full knockout mouse model of IP3R2 leads to abolishment of spontaneous and Gq-GPCR linked Ca^2+^ increases in astrocytes (Petravicz et al., 2008). However, this mouse line is unsuited to behavioral testing due to potential issues with altered Ca^2+^ signaling in physiological processes outside the CNS (Li et al., 2005; Lipp et al., 2009). We therefore generated a conditional knockout mouse model to restrict the deletion primarily to GFAP^+^ glial cell populations. To determine the effective level of recombination and assess the number of astrocytes lacking IP3R2-mediated signaling, immunohistochemistry for IP3R2 was performed on IP3R2 cKO mice (*n* = 2) and compared to littermate controls (*n* = 2). Serial sections were stained for IP3R2 and colocalized with GFAP for astrocytes to assess extent of recombination. In control sections, all GFAP positive cells were also positive for IP3R2 (Figure 1A). In IP3R2 cKO sections, IP3R2 colocalization with GFAP was significantly reduced in all brain sections examined. In the hippocampus, there was an 82% reduction in the number of GFAP-positive astrocytes expressing IP3R2 (*p* = 0.007, Figures 1A,B). Similar reductions were found in the cortex (90% reduction, *p* = 0.0001) and in the substantia nigra (95% reduction, *p* = 0.0002). Further, we see no evidence for reactive gliosis or alterations to astrocyte morphology based on GFAP immunohistochemistry (data not shown).

To assess functional loss of IP3R2-mediated Ca^2+^ signaling in astrocytes, Ca^2+^ imaging studies were conducted on acute brain tissue preparations from IP3R2 cKO mice. Astrocytes respond to multiple families of GPCRs including glutamatergic (Schools and Kimelberg, 1999), cholinergic (Chen et al., 2012) and histaminergic (Shelton and McCarthy, 2000) systems [though the presence of Gq coupled mGluRs in astrocytes has recently been questioned, see Sun et al. (2013)]. We applied an agonist mixture to target all three of these Gq GPCR pathways to engage multiple Gq coupled GPCRs and elicit a maximal response. Imaging in the hippocampus found a significant reduction (*p* < 0.001) in the number of astrocytes from the IP3R2 cKO models able to respond with cytosolic Ca^2+^ increases upon application of Gq GPCR agonists (coapplication of 10 μM DHPG, 10 μM carbachol, 10 μM histamine) known to elicit Ca^2+^ increases (Figures 1C,D: Control, 84 of 97 (86.5%) cells responding, 8 animals; IP3R2 cKO, 2 of 63 (3.2%) cells responding, 7 animals). These findings agree with previously published work demonstrating that IP3R2 is necessary to generate cytosolic and process-localized Ca^2+^ responses in astrocytes, but not neurons, in acute slices and *in vivo* in several brain regions including hippocampus, cerebellum, visual, and somatosensory cortexes (Petravicz et al., 2008; Di Castro et al., 2011; Panatier et al., 2011; Takata et al., 2011, 2013; Navarrete et al., 2012; Tamamushi et al., 2012; Nizar et al., 2013; Haustein et al., 2014). Additionally, similar results using the IP3R2 cKO were observed in the visual cortex for both recombination efficiency and lack of stimulus induced Ca^2+^ signals (Chen et al., 2012). The data presented above, combined with the previous finding provide strong evidence that the GFAP-Cre system is highly effective at recombining the floxed IP3R2 allele, resulting in the abolishment of IP3R2-dependent Ca^2+^ signaling in astrocytes across multiple brain regions.

### Behavioral testing of the IP3R2 cKO mouse model

Tests were performed to assess the effect of eliminating astrocytic Gq-GPCR mediated Ca^2+^ responses on several categories of behavior. Categories included anxiety and depression (elevated plus maze, suspended tail hang), motor function (open field activity, accelerating rotarod), sensory function (acoustic startle test) and learning and memory (Morris water maze). IP3R2 cKO mice and littermate controls were housed together and tested in a blinded manner to eliminate tester bias.

#### Anxiety and depression

**Figure 2:**
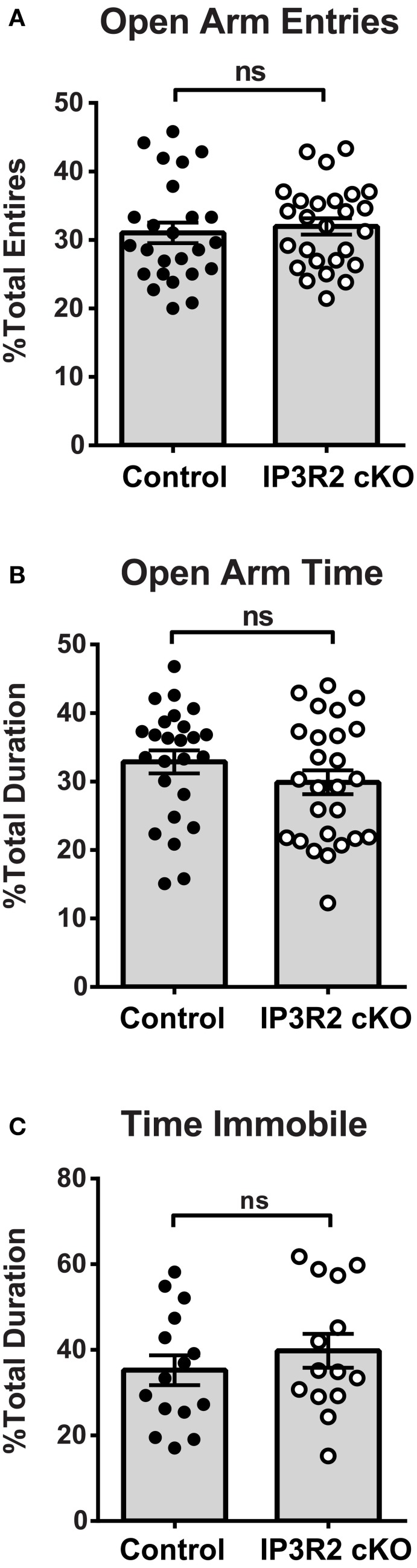
**Anxiety and depressive behaviors are not affected in the IP3R2 cKO**. **(A,B)** Anxiety-like behavior in the elevated plus maze is unaffected in IP3R2 cKO mice. IP3R2 cKO (*n* = 26, clear circles) and littermate control (*n* = 25, black circles) mice are not significantly different for open arm entries (*p* = 0.62) or percent duration spent in open arms (*p* = 0.22) in the elevated plus maze. **(C)** Depressive-like behaviors measured by tail suspension are unaffected in IP3R2 cKO mice. No significant difference (*p* = 0.39) was observed between the IP3R2 cKO (*n* = 14, clear circles) and controls (*n* = 15, black circles) in the suspended tail hang test. Error bars indicate s.e.m.

The plus maze is an extensively used test of anxiety and exploratory behavior in mice (Dawson and Tricklebank, 1995). Plus maze analysis of IP3R2 cKO mice (*n* = 26) and controls (*n* = 25) found no significant alterations in both the percentage of open entries (Figure 2A: Control, 31.0 ± 1.5%; IP3R2 cKO, 31.9 ± 1.2%; *p* = 0.62, *t* = 0.50, *df* = 49) or percent open time (Figure 2B: Control, 32.8 ± 1.7%; IP3R2 cKO, 29.9 ± 1.6%; *p* = 0.22, *t* = 1.24, *df* = 49) when compared to controls. As an opposing behavioral measure, testing for depressive-like behaviors using the tail suspension test (Crowley et al., 2004) also showed no significant difference between IP3R2 cKO and controls (Figure 2C: Control, 35.2 ± 3.5%, *n* = 15; IP3R2 cKO, 39.7 ± 3.9%, *n* = 14; *p* = 0.39, *t* = 0.86, *df* = 27). These results indicate that lack of IP3R2-mediated Ca^2+^ signals in astrocytes has no significant impact on these behaviors.

#### Motor activity and sensorimotor gating

**Figure 3:**
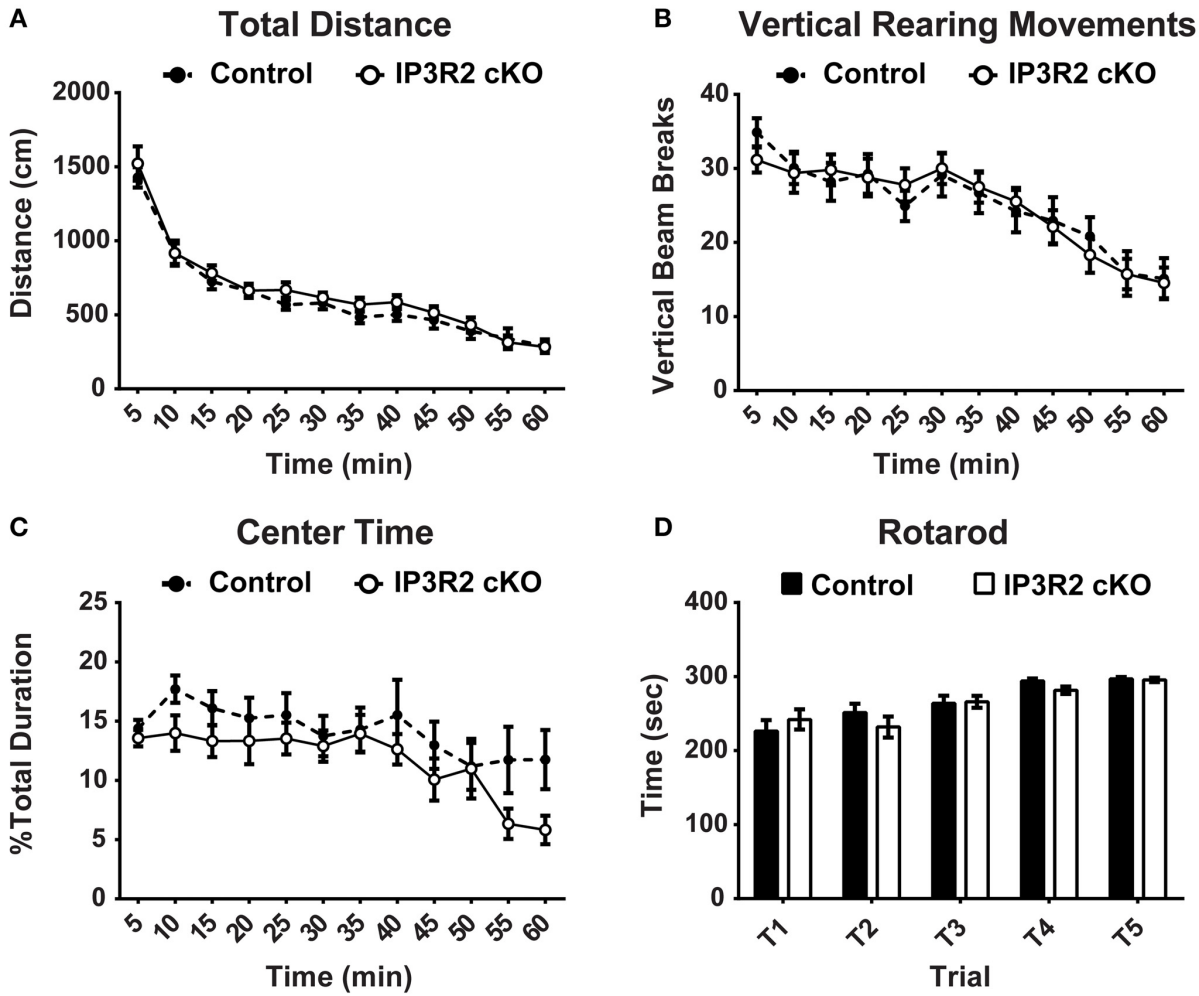
**Motor function and exploratory behavior are unaffected in the IP3R2 cKO**. **(A,B)** Spontaneous locomotor activity and exploratory behavior measured in the open field activity task was unaffected in the IP3R2 cKO. IP3R2 cKO (clear circles, *n* = 26) mice were not significantly different (*p* = 0.43) from control mice (black circles, *n* = 26) in spontaneous motor activity measured by total distance in open field testing or vertical rearing movements. **(C)** Anxiety measured by percentage of time spent in or moving through the center of the activity box was also not significantly different (*p* = 0.14). **(D)** Motor coordination and learning over a 2-day, 5 trial testing period using an accelerating rotarod was not significantly different (*p* = 0.70) between IP3R2 cKO (clear bars, *n* = 26) and control animals (black bars, *n* = 26).

Motor activity elicits Ca^2+^ increases in both Bergmann glia and astrocytes of the cerebellum *in vivo* (Nimmerjahn et al., 2009). Further, Ca^2+^ fluxes in cerebellar Bergmann glia and astrocytes are largely dependent on IP3R2 (Tamamushi et al., 2012). We conducted two different tests to determine if motor function in IP3R2 cKO mice was altered. Spontaneous motor activity was measured using a 1-h open field test. No significant main genotype effect [*p* = 0.43, *F*_(1, 50)_ = 0.63] was found between IP3R2 cKO (*n* = 26) and controls (*n* = 26) for total distance traveled in the open field (Figure 3A). Further, vertical rearing movement between IP3R2 cKO and controls was not significantly different (Figure 3B). Lastly, a secondary measure of anxiety can be determined from open field testing by calculating the percentage of time mice spend crossing into and through the center region of the activity arena. No significant main genotype effect [*p* = 0.14, *F*_(1, 50)_ = 2.19] was found between control and IP3R2 cKO for percent center time in the activity arena (Figure 3C). Therefore, we find no indication of an alteration in spontaneous motor activity in IP3R2 cKO animals.

**Figure 4:**
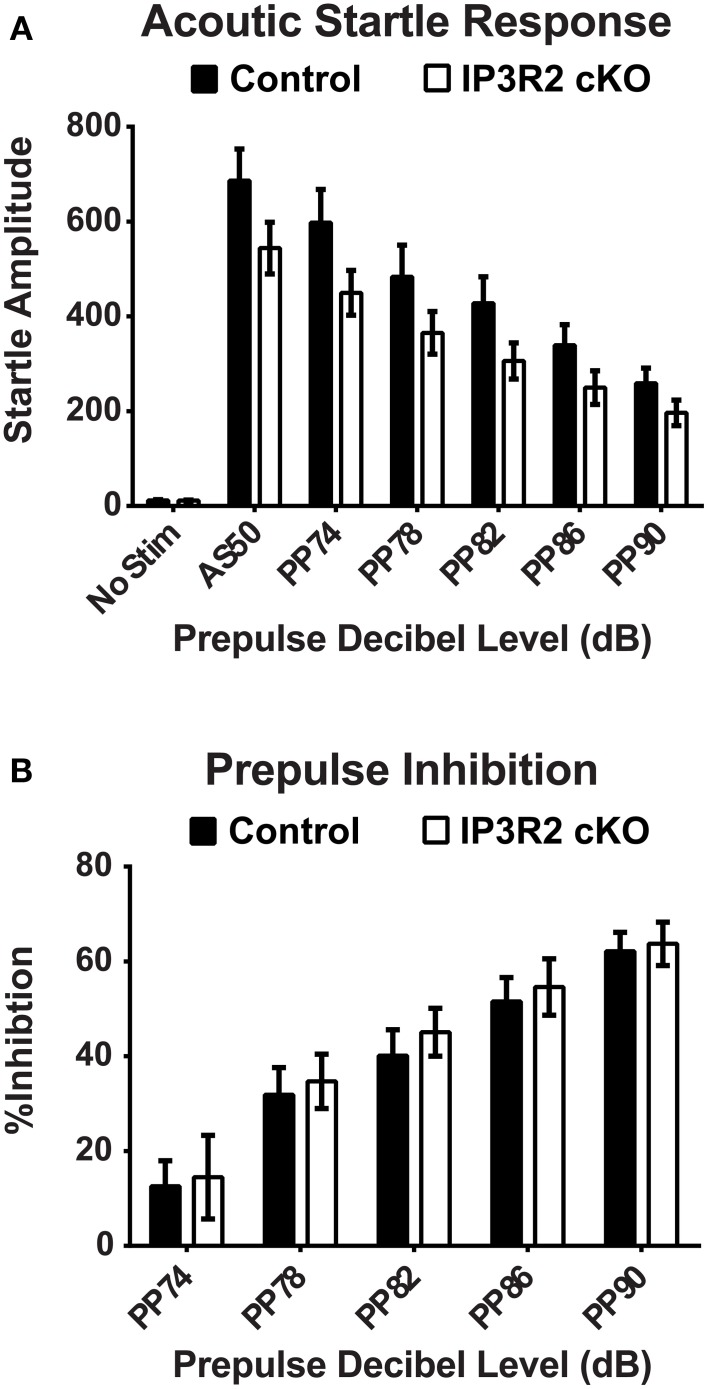
**Sensory motor gating and prepulse inhibition are unaltered in the IP3R2 cKO. (A)** Sensory motor gating measured by response amplitude to acoustic startle with differing prepulses displayed no significant difference (*p* = 0.1) between IP_3_R2 cKO mice (clear bars, *n* = 26) compared to controls (black bars, *n* = 25). **(B)** Prepulse inhibition was not significantly different (*p* = 0.7) between IP3R2 cKO (clear bars, *n* = 26) and control mice (black bars, *n* = 25). Error bars indicate s.e.m.

The accelerating rotarod is commonly used as a test of motor coordination and learning in mice (Paylor et al., 1998; Hossain et al., 2004). Repeated trials over the course of 2 days with an accelerating rotarod paradigm found no significant main genotype effect [*p* = 0.70, *F*_(1, 50)_ = 0.15] in the latency to fall off the rotarod or ability to improve trial by trial performance between IP3R2 cKO (*n* = 26) and control (*n* = 26) mice (Figure 3D). Lastly, sensory motor gating was tested using the acoustic startle response (ASR) with a pre-pulse inhibition (PPI) testing paradigm. No significant main genotype effect [*p* = 0.1, *F*_(1, 49)_ = 2.94] on the ASR amplitude in IP3R2 cKO mice compared to controls (Figure 4A: Control, *n* = 25; IP3R2 cKO, n = 26), with *post-hoc* tests reporting no significant difference between IP3R2 cKO and control mice at any stimulus intensity (No Stim: *p* > 0.99; AS50: *p* = 0.26; PP74: *p* = 0.17; PP78: *p* = 0.5; PP82: *p* = 0.45; PP86: *p* > 0.99; PP90: *p* > 0.99). Further, no significant genotype effect [*p* = 0.7, *F*_(1, 49)_ = 0.148] was observed upon calculation of the ASR pre-pulse inhibition (Figure 4B) indicating no difference between the cohorts. Collectively, lack of IP3R2 mediated Ca^2+^ signaling in astrocytes has no significant effect on motor function or motor learning.

#### Learning and memory

**Figure 5:**
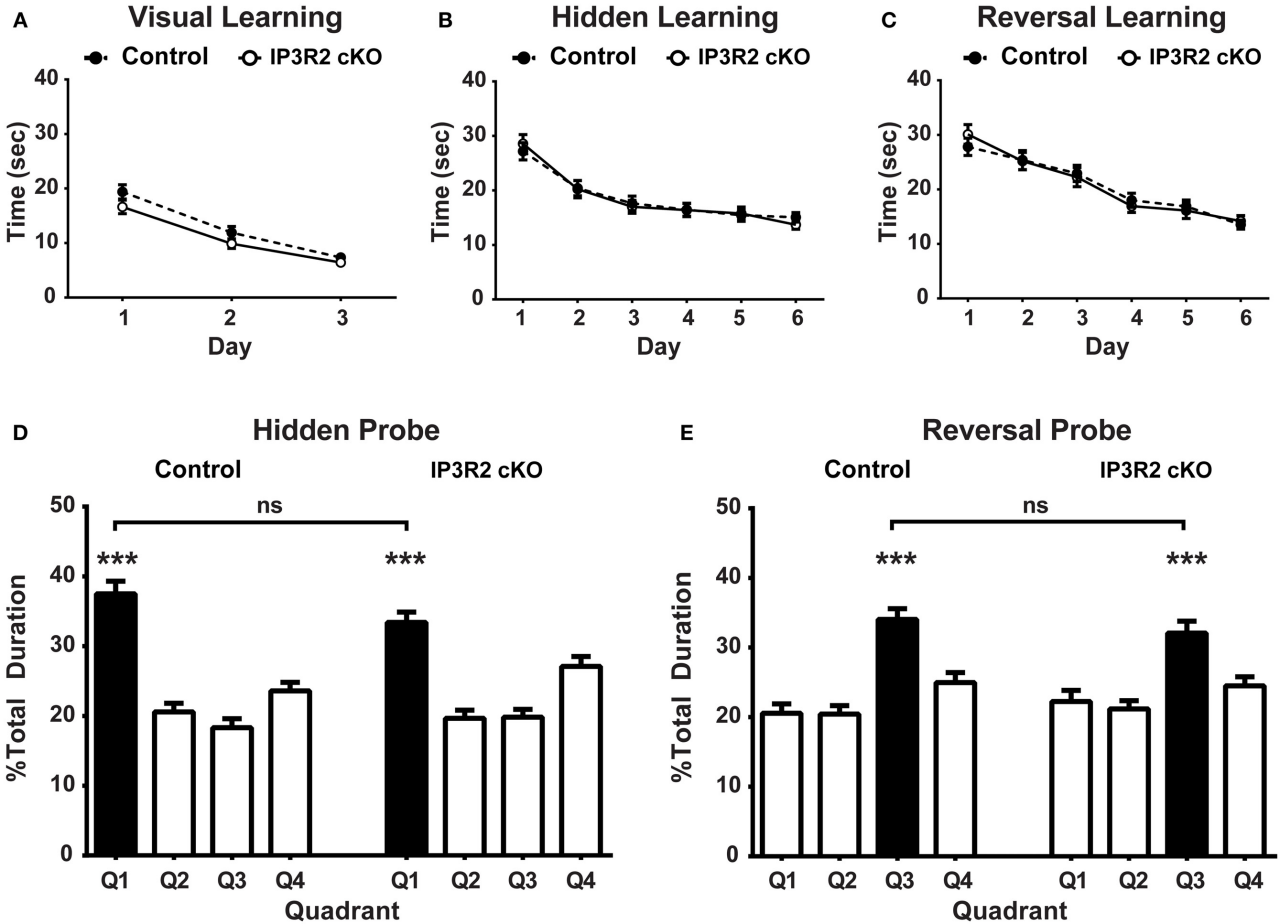
**Morris water maze for learning and memory are unaffected in the IP3R2 cKO**. **(A)** Training for the visually identified location of the escape platform was not significantly different (*p* = 0.08) between IP3R2 cKO mice (clear circles, *n* = 46) and control mice (solid circles, *n* = 53). **(B)** Hidden platform training over the course of 6 days showed that IP3R2 cKO mice displayed no significant difference in acquisition of the location of hidden platform compared to controls (*p* = 0.92). **(C)** Reversal of acquisition and acquisition of the relocated platform location training over the course of 6 days displays no significant difference between IP3R2 cKO and controls (*p* = 0.97). **(D)** Both IP3R2 cKO and control mice present a significant preference (IP3R2 cKO, *p* ≤ 0.0001; control *p* < 0.0001) for the target quadrant (Q1) in the probe trial of the hidden platform acquisition task. Direct comparison of Q1 preference is not significantly different (*p* = 0.57) between the two groups. **(E)** Both IP3R2 cKO (*p* < 0.0001) and control mice (*p* < 0.0001) display a significant preference for the target quadrant (Q3) in the probe trial of the reversal hidden platform task. Direct comparison of Q3 preference is also not significantly different (*p* = 0.99) between the two groups. Error bars indicate s.e.m. ^***^ *p* < 0.001.

Astrocyte IP3R2- mediated, Ca^2+^-dependent release of gliotransmitters is believed to be critical to synaptic mechanisms underlying learning and memory (Pascual et al., 2005; Serrano et al., 2006; Henneberger et al., 2010; Chen et al., 2012; Han et al., 2012; Min and Nevian, 2012; Navarrete et al., 2012). The Morris water maze (MWM) is a highly validated and accepted behavioral correlate of learning and memory involving several brain regions, but is primarily a hippocampus-based test in the paradigm used (Schwegler et al., 1988; Tsien et al., 1996; Logue et al., 1997; D'hooge and De Deyn, 2001; Florian and Roullet, 2004; Vorhees and Williams, 2006). In order to probe changes to learning and memory *in vivo*, we conducted MWM testing of IP3R2 cKO mice. Visual platform training of IP3R2 cKO (*n* = 46) and control mice (*n* = 53) found no significant main genotype effect [*p* = 0.08, *F*_(1, 97)_ = 3.235] in the latency to find the visually cued escape platform over 3 days of training (day 1: *p* = 0.17; day 2: *p* = 0.47; day 3: *p* > 0.99), indicating all mice were motivated to swim and could visually recognize the location of the escape platform (Figure 5A). The next stage of the MWM testing, acquisition of the hidden escape platform location also revealed no significant main genotype effect [*p* = 0.92, *F*_(1, 97)_ = 0.009) for escape latency over the course of 6 days of training between genotypes (Figure 5B). Two hours after the behavioral cohort (both control and IP3R2 cKO mice) met criteria for having learned the hidden acquisition task (defined as average cohort escape time of 15 s), mice were tested with a single 1 min probe trial with the platform removed. Both control [*p* < 0.0001, *F*_(2.1, 111.4)_ = 27.97] and IP3R2 cKO mice [*p* < 0.0001, *F*_(2.4, 109.1)_ = 19.25] exhibit a significant preference for the target quadrant, defined as the location of the platform prior to removal (Figure 5D). Comparison between the two cohorts found no significant difference in their performance [*p* = 0.57, *F*_(1, 97)_ = 0.33] and no significant difference between IP3R2 cKO and control animal populations for the target quadrant (4.09 + 1.9 mean difference; *p* = 0.14) or the off-target quadrants (Figure S1). These findings indicate that removal of astrocytic IP3R2-dependent Ca^2+^ signaling has no apparent effect upon a hippocampal-based learning and memory behavior.

To test for cognitive flexibility and prefrontal cortex impairment, we next conducted the reversal of acquisition paradigm for the MWM. During reversal, the hidden escape platform is relocated to the opposite quadrant from acquisition and the subjects are trained to learn the new location as a test of behavioral flexibility. No genotype effect [*p* = 0.97, *F*_(1, 97)_ = 0.001] was found in the ability of the IP3R2 cKO mice to acquire the new platform location compared to controls (Figure 5C). The reversal of acquisition probe trial found that both the IP3R2 cKO [*p* < 0.0001, *F*_(2.2, 100.3)_ = 12.80] and control mice [*p* < 0.0001, *F*_(2.3, 121.6)_ = 15.97] exhibit a preference for the new target quadrant (Figure 5E). Comparison between the two cohorts found no significant difference in their performance [*p* = 0.38, *F*_(1, 97)_ = 0.78] and no significant difference between the two populations for the target quadrant (1.96 + 2.0 mean difference; *p* > 0.99) or off-target quadrants (Figure S2). Collectively, we find no evidence that lack of IP3R2-mediated Ca^2+^ signaling in astrocytes affects the ability of IP3R2 cKO animals to successfully learn, remember, and be retrained on the spatial location of an escape platform in MWM.

## Discussion

Astrocytic IP3R2-mediated Ca^2+^-dependent signaling has been implicated in the modulation of nearly every aspect of neuronal synaptic transmission, as well as more general brain functions such as cerebrovascular control and metabolism (for reviews see Haydon and Carmignoto, 2006; Fellin et al., 2006; Gordon et al., 2007; Agulhon et al., 2008, 2012; Perea et al., 2009; Allaman et al., 2011; Petzold and Murthy, 2011; Blutstein and Haydon, 2013). Evidence for modulation of presynaptic fidelity (Navarrete and Araque, 2010; Panatier et al., 2011), heterosynaptic depression and LTP (Pascual et al., 2005; Chen et al., 2013), and postsynaptic NMDA receptor function (Parri et al., 2001; Perea and Araque, 2005) by astrocytes has accumulated rapidly. Further, it has been proposed that IP3R2-mediated Ca^2+^-dependent signaling is the basis of gliotransmission and an integral component of a wide range of physiological processes including sleep (Halassa et al., 2009; Hines and Haydon, 2013; Nadjar et al., 2013), functional hyperemia (Filosa et al., 2004; Mulligan and Macvicar, 2004; Girouard et al., 2010; He et al., 2012), metabolism (Zheng et al., 2013), and synaptic mechanisms underlying learning and memory formation (Pascual et al., 2005; Serrano et al., 2006; Henneberger et al., 2010; Han et al., 2012; Min and Nevian, 2012; Navarrete et al., 2012; Chen et al., 2013). With the realization that IP3R is primarily, if not exclusively responsible for all GPCR elicited Ca^2+^ fluxes in astrocytes (Petravicz et al., 2008; Haustein et al., 2014; Kanemaru et al., 2014) and the availability of IP3R2 cKO mice, it became possible to determine if astrocytic IP3R2-mediated Ca^2+^ signals were important in behavior. Given the wide range of neuronal and cerebrovascular mechanisms that are hypothesized to be modulated by IP3R2-mediated Ca^2+^ signaling in the brain, the lack of a broadly based behavioral study of the consequences of blocking this signaling pathway represented a fundamental void in our knowledge of astrocyte physiology. This behavior-based approach to studying the role of astrocyte Ca^2+^ signaling in physiology is in marked contrast to the majority of studies that rely on cultured astrocytes, acute slice or *in vivo* electrophysiology and Ca^2+^ imaging. Our study is the first behavioral analysis on the effect of silencing the proposed major pathway involved in neuronal-astrocyte communication specifically in astrocytes to date. We demonstrate that on the functional level of whole animal behavior, there is no evidence that astrocytic IP3R-mediated, Ca^2+^-dependent signaling (the proposed necessary and sufficient component of gliotransmission) plays a significant role in modulating the neuronal circuits and synaptic plasticity underlying the behaviors tested. Findings presented here, combined with previous electrophysiological studies (Fiacco et al., 2007; Petravicz et al., 2008; Agulhon et al., 2010), suggest that Ca^2+^ dependent gliotransmission is not playing a prominent role in neuronal circuits, synaptic transmission and plasticity *in situ* or *in vivo* in normal physiological states.

It is well known that astrocytes are capable of responding to sensory evoked neuronal activity *in vivo* with increases in intracellular Ca^2+^ (Chen et al., 2012; Zhao et al., 2012; Ding et al., 2013; Takata et al., 2013; Bonder and McCarthy, 2014; Paukert et al., 2014; Perez-Alvarez et al., 2014). Less understood *in vivo* is the downstream results of the activation of this signaling pathway in astrocytes. The majority of literature concerning the role of astrocyte Ca^2+^ signaling suggests that they play a major role in modulating synaptic activity, control of cerebral blood flow in response to neuronal activity, and are therefore likely to influence behavior. Paukert et al. (2014) recently reported that astrocyte in the primary visual cortex and the cerebellum demonstrated enhanced Ca^2+^ responsiveness to sensory-evoked stimuli during forced locomotion that was dependent on noradrenergic signaling, providing evidence that astrocytes are capable of responding to the behavioral state of the animal. However, the authors propose that due to timing of the onset of astrocyte Ca^2+^ responses, it is unlikely to be involved in the initial sensory-stimulus response of cortical neurons but rather in longer time scale processing or shifts in attentional states. Therefore, it is likely that astrocytes are involved primarily in modulating the dynamic range of neuronal activity to optimize performance *in vivo* through their Ca^2+^ signaling and downstream pathways (Wang et al., 2012) rather than direct engagement in sensory information processing. Impairment of this function to respond to sensory evoked stimuli may not impede neuronal networks from performing the necessary computation underlying behavior in a significant manner. If this is the case, then one could expect subtle or no impact on behavior, which is the result of our study.

Artificial activation of astrocytes and Bergmann glial cells via channelrhodopsin (ChR) were recently reported to cause alterations in optokinetic behavior in awake, behaving mice (Sasaki et al., 2012). These findings lend support to the concept that astrocytes are integral components of behavioral circuits in the brain. However, there are several relevant caveats to the Sasaki study. First, Bergmann glia have several fundamental differences compared to astrocytes: they are morphologically distinct from astrocytes, express multiple IP3 receptors, detect synaptic activity at cerebellar synapses primarily through activation of Ca^2+^ permeable AMPA receptors as well as Gq-linked GPCRs, and are known to ectopically release glutamate upon activation of these Ca^2+^ permeable AMPARs (Burnashev et al., 1992; Muller et al., 1992; Tamamushi et al., 2012). This makes Bergmann glia functionally very different in how they respond to neuronal activity when compared to astrocytes. Second, optogenetic stimulation bypasses endogenous signaling pathways in glial cells, and may lead to non-physiological alterations of neuronal activity. Channelrhodpsins are non-selective cation channels that pass sodium, protons, and weakly conduct calcium (Nagel et al., 2003; Lorenz-Fonfria and Heberle, 2014) making ChRs a very unselective tool to activate glial cells. For example, activation of ChRs leads to a large inward current into Bergmann glia in Sasaki et al. (2012) that was partially attributed to potassium fluxes, where other studies report ChR-mediated depolarization with Ca^2+^ increases (Gourine et al., 2010). Due to the nature of ChRs, artificial stimulation with optogenetics is akin to Ca^2+^ or IP3 uncaging in astrocytes and Bergmann glia which is now recognized to not accurately reflect physiological levels of stimulation (Fiacco et al., 2007; Wang et al., 2013). Lastly, the release of glutamate from Bergmann glial cells in Sasaki et al. (2012) was found to occur through a DIDS-sensitive anion channel and does not involve Ca^2+^ dependent release from internal stores, making it difficult to directly compare their findings to ours. This is a fundamentally different mechanism than that underlying the current definition of gliotransmission from astrocytes and therefore may not be affected by deletion of IP3R2 from astrocytes and Bergmann glia. This highlights the potential pitfalls in attributing physiological roles for astrocyte function when using non-physiological stimulation and the interpretation of results with these methods.

Two recent reports found evidence for behavioral alterations in mice where IP3R-dependent Ca^2+^ increases in astrocytes were altered. Tanaka et al. (2013) reported that the expression of an inducible “IP3-sponge” construct attenuated, but not completely blocked IP3R-mediated Ca^2+^ signaling in astrocytes. When this mouse model was tested in MWM, mice with attenuated Ca^2+^ signaling displayed a significant reduction in the time spent in the target area when compared to controls, but still were able to successfully learn and remember the location of the platform. This alteration was attributed not to a blockade in IP3R-mediated Ca^2+^-dependent gliotransmission, but rather a retraction of glial processes and removal of glutamate transporters from around synapses. Importantly, no alterations to LTP, LTD or other hippocampal synaptic plasticity based mechanisms related to learning and memory using several conventional stimulation protocols in acute slice recordings were observed in these mice calling into question the relevance of IP3R-mediated Ca^2+^ signaling in modulation of synaptic plasticity. Furthermore, no alterations to open field activity or elevated plus maze were detected in the study, in agreement with our findings. Given the difference in techniques for blocking astrocyte Ca^2+^ signaling between the Tanaka study and our present study, it is difficult to know the basis for the difference in our findings in regards to the Morris water maze. The Tanaka study found that altered, but not abolished, IP3R-mediated Ca^2+^ signaling resulted in removal of astrocyte processes from synapses and reduced glutamate reuptake capacity underlie their behavioral phenotypes. However, given that the germ-line IP3R2 KO mouse model had no alterations in ambient glutamate and tonic NMDA receptor activation (Petravicz et al., 2008) indicative of impaired glutamate reuptake it is unlikely that retraction of astrocyte processes and removal of glutamate transporters is occurring in the IP3R2 cKO mouse model. Interestingly, it was recently found that in the germline IP3R2 KO, astrocytic processes lack the ability to retract from synapses in response to neuronal activity both *ex vivo* and *in vivo* (Perez-Alvarez et al., 2014), lending support to this stance. In a separate study, Cao et al. (2013) reported alterations in depressive-like behaviors in a full germ-line IP3R2 KO model using the forced swim test, but no alterations to open field or elevated plus maze. Our testing for depressive behaviors using the tail suspension test found no such alterations. IP3R2 is expressed in a number of cells outside the CNS (Futatsugi et al., 1998; Grayson et al., 2004; Li et al., 2005; Cruz et al., 2010) complicating the interpretation of experiments using the full IP3R2 KO. For example, atrial myocytes have altered responses to neurohumoral stimulation, which may introduce unidentifiable alterations to behavior (Li et al., 2005). Further, the Cao et al. (2013) study employed multiple mouse models with varying background strains, making it difficult to place their findings in context with our own work due to strain-specific differences in behavior.

There are several potential explanations as to why we do not see behavioral changes following the removal of astrocyte IP3R2-dependent calcium fluxes. First, the IP3R2 cKO mouse model blocks astrocyte IP3R-mediated Ca^2+^ signals starting relatively early in development. It is possible that neuronal circuits rewire to compensate for the loss of astrocyte Ca^2+^ signaling during development obscuring the role of astrocyte Ca^2+^ fluxes in behavior; this seems unlikely given the breath of behaviors unaffected by the loss of astrocyte Ca^2+^ signaling. It is also possible that intrinsic plasticity mechanisms (e.g., homeostatic scaling of synapses) in neurons compensates for the loss of astrocyte IP3R2 dependent Ca^2+^ fluxes. While this also seems unlikely given that the absence of IP3R2-dependent Ca^2+^ signaling in astrocytes has no demonstrable effect on basal synaptic transmission or synaptic plasticity (Agulhon et al., 2010), it formally remains a possibility. Further, given the range of behavior tests used and the brain regions tested, for compensation to occur on such a broad scale across different components of the complex circuits governing behavior to result in no observable difference is equally unlikely. A more plausible explanation is that fluxes in astrocyte Ca^2+^ reliant on IP3R2 are not playing a major role in the behavioral tests performed. Second, on the level of intracellular signaling, it is possible that changes in alternate pathways compensate for the loss of astrocytic Ca^2+^ signaling. This seems highly doubtful given that alternate second messenger pathways are unlikely to compensate for the loss of Ca^2+^ signaling. While alternate Ca^2+^ sources (e.g., plasma membrane calcium channel mediated increases) have recently been implicated in regulating basal synaptic transmission (Shigetomi et al., 2012), it is unlikely they would be capable of compensating for the loss of GPCR-mediated, IP3R-dependent Ca^2+^ signaling which are currently hypothesized to drive gliotransmission *in situ* (Perea et al., 2009). The activity of these channels in astrocytes does not appear to be regulated by neuronal activity, and therefore is unlikely to be involved in gliotransmission. Further, no detectable Ca^2+^ responses are observed in hippocampal astrocytes from IP3R2 KO mice following intense stimulation of the Schaffer collateral pathway. It is more plausible that GPCR mediated increases in astrocytic Ca^2+^ serve alternate roles not involved in modulating the primary behavioral tests performed in our studies. It also seems likely that as additional task specific behavioral tests in IP3R2 cKO mice are performed, phenotypes will be observed. Additionally, studies utilizing more regionally restricted or inducible knockout mouse models may reveal roles for astrocytes in the modulation of animal behavior.

The IP3R2 cKO mouse model has been used in one previous publication, in which Chen et al. (2012) explored the role of astrocyte Ca^2+^ signaling in cholinergic potentiation in the primary visual cortex of anesthetized mice. The authors reported a lack of cholinergic potentiation of excitatory neuron responses to paired nucleus basalis stimulation and specific visual orientations. The findings of Chen et al. (2012) suggest alterations to visual cortex neuronal circuits that may affect visual processing and therefore spatial navigation. Our present behavior data find no alterations to spatial navigation in the MWM. Cholinergic modulation of primary visual cortex (V1) neuronal responses involving astrocytes may either not be engaged in our behavioral task, or more likely are not of sufficient strength to result in detectable behavioral changes. It is important to note that Chen et al. (2012) did not conduct behavioral test of any kind to confirm whether potentiation in visual responses of a subset of neurons would be sufficient to produce any behavioral change.

The hypothesized physiological role of the IP3-dependent increases in astrocyte Ca^2+^ has recently undergone scrutiny, primarily due to the advent of the IP3R2 KO mouse model. Several independent investigators have published evidence supporting our previous findings that IP3R2 is the primary mediator of astrocytes Ca^2+^ signaling, with universal agreement that this mouse model blocks GPCR/IP3-based Ca^2+^ signaling (both spontaneous and stimuli evoked) in astrocytes (Chen et al., 2012; Navarrete et al., 2012; Thrane et al., 2012; Wang et al., 2012; Nizar et al., 2013; Haustein et al., 2014; Kanemaru et al., 2014). Recently, IP3R2 independent Ca^2+^ signaling pathways in astrocytes have been identified both *in situ* (Haustein et al., 2014) and *in vivo* (Shigetomi et al., 2012, 2013; Kanemaru et al., 2014). Importantly, these IP3R2 independent Ca^2+^ fluxes in the cited studies above are not regulated by neuronal activity and therefore are not likely to be involved in regulating synaptic transmission in response to neuronal activity. Two of these studies (Haustein et al., 2014; Kanemaru et al., 2014) utilized germline IP3R2 KO mice expressing genetically encoded Ca^2+^ indictors to show that astrocytes display microdomain Ca^2+^ increases that are not reliant on IP3 receptors. TRPA1 channels dependent Ca^2+^ influxes, which are not regulated by neuronal activity, regulate the release of D-serine to modulate hippocampal LTP (Shigetomi et al., 2013), a function previous ascribed to neuronal activity dependent release from astrocytes via IP3R-mediated Ca^2+^ signaling (Henneberger et al., 2010). Additionally, IP3R2-mediated Ca^2+^ increases were recently reported to not be required for functional hyperemia (Nizar et al., 2013; Takata et al., 2013; Bonder and McCarthy, 2014); these *in vivo* studies call into question a large number of *in situ* studies reporting that IP3R dependent increases in astrocyte Ca^2+^ modulate blood flow. These emerging data support the view that IP3 receptor mediated Ca^2+^ release from internal stores may not be involved in the acute modulation of neuronal activity, and that IP3R-mediated Ca^2+^ signaling is serving alternative roles that remain largely unknown.

In conclusion, our findings indicate that astrocytic IP3R-mediated Ca^2+^ dependent signaling and its downstream pathways are not major modulators of the physiological pathways governing behavior. The striking lack of major biological behavioral phenotypes in astrocyte IP3R2 cKO is in marked contrast to other astrocyte-specific genetic manipulations where strong phenotypes have been observed (Frisch et al., 2003; Theis et al., 2003; Abu-Ghanem et al., 2008; Kiryk et al., 2008). Collectively, these findings suggest that astrocytic Ca^2+^ signaling through IP3Rs may serve, as yet, unknown roles. Our study extends our previous findings (Fiacco et al., 2007; Petravicz et al., 2008; Agulhon et al., 2010) and strongly suggests that the concept that astrocytes are significant modulators of synaptic activity and plasticity via the IP3R-mediated Ca^2+^ dependent release of gliotransmitters needs to be reconsidered.

### Conflict of interest statement

The authors declare that the research was conducted in the absence of any commercial or financial relationships that could be construed as a potential conflict of interest.
