# Supplementary material for: Astrocyte IP3R2-dependent Ca2+ signaling is not a major modulator of neuronal pathways governing behavior
Source: Front Behav Neurosci. 2014 Nov 12;8:384. doi: 10.3389/fnbeh.2014.00384 (PMC4228853; doi:10.3389/fnbeh.2014.00384)
Supplement: Supplementary file 1 [file DataSheet1.DOCX]

***Supplementary Material***

**Astrocyte IP3R2-dependent Ca2+ signaling is not a major modulator of neuronal pathways governing behavior.**

**Jeremy Petravicz^1,†^, Kristen M. Boyt^2^, Ken D. McCarthy^1,2,*^**

**^1^Curriculum in Neurobiology, University of North Carolina at Chapel Hill, Chapel Hill, North Carolina 27599.**

**^2^Department of Pharmacology, University of North Carolina at Chapel Hill, Chapel Hill, North Carolina 27599**

**^†^Current affiliation: Picower Institute for Learning and Memory, Department of Brain and Cognitive Sciences, Massachusetts Institute of Technology, Cambridge, MA 02139.**

*** Correspondence:** Ken D. McCarthy, CB # 7365, UNC-CH School of Medicine, Chapel Hill, NC 27599-7365, kdm@ad.unc.edu

1. **Supplementary Data**


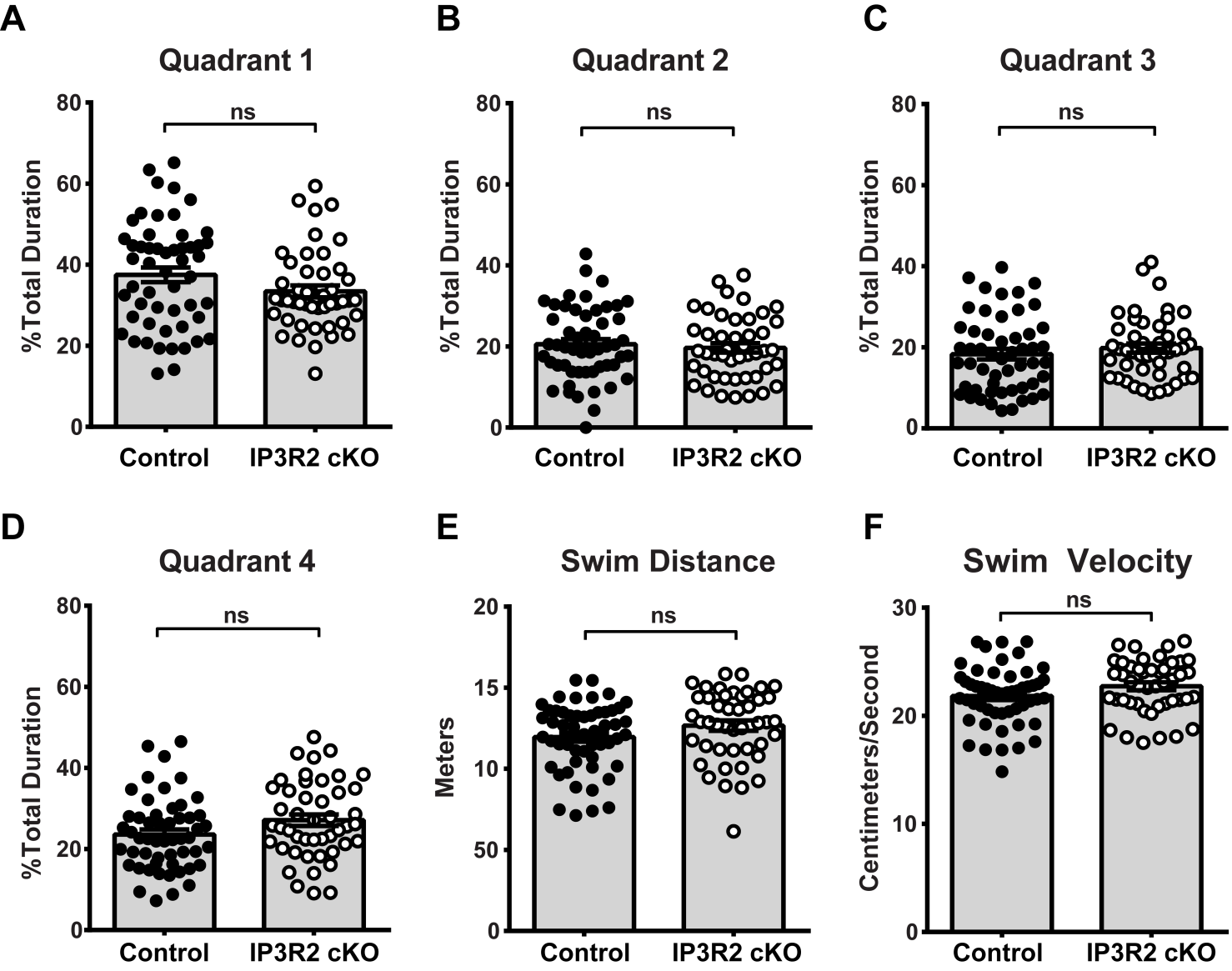


**Supplementary Figure 1. Statistical comparison of individual quadrant durations from the hidden probe trial of the MWM.** (A-D) Individual quadrants scores for IP3R2 cKO (clear circles, n = 49) and control (black circles, n = 53) are plotted as both the mean (bar graph of mean with SEM) and a scatter plot to show population values. Each dot represents one animal. Post-hoc testing after two-way repeated measures ANOVA found no significant differences for Quadrant 1 (p = 0.14), Quadrant 2 (p > 0.99), Quadrant 3 (p > 0.99) or Quadrant 4 (p = 0.28). (E) Total distance swam during the one-min probe trial in centimeters is not significantly different between IP3R2 cKO and control (Student’s t-test: p = 0.1, t = 1.66, df = 97). (F) Swim velocity during the one-minute probe trials measured in centimeters/second is also unaffected (p = 0.1, t = 1.71, df = 97). Data is presented as mean + SEM.
